# Transthyretin derived amyloid deposits in the atrium and the aortic valve: insights from multimodality evaluations and mid-term follow up

**DOI:** 10.1186/s12872-023-03319-3

**Published:** 2023-06-01

**Authors:** Atsushi Okada, Takashi Kakuta, Naoki Tadokoro, Emi Tateishi, Yoshiaki Morita, Takeshi Kitai, Makoto Amaki, Hideaki Kanzaki, Keiko Ohta-Ogo, Yoshihiko Ikeda, Satsuki Fukushima, Tomoyuki Fujita, Kengo Kusano, Teruo Noguchi, Chisato Izumi

**Affiliations:** 1 Department of Cardiovascular Medicine, National Cerebral and Cardiovascular Center, 6-1 Kishibe-Shimmachi, Suita, Osaka 564-8565 Japan; 2 Department of Cardiovascular Surgery, National Cerebral and Cardiovascular Center, Suita, Osaka Japan; 3 Department of Radiology, National Cerebral and Cardiovascular Center, Suita, Osaka Japan; 4 Department of Pathology, National Cerebral and Cardiovascular Center, Suita, Osaka Japan

**Keywords:** Amyloidosis, Cardiac amyloidosis, Amyloid cardiomyopathy, Extraventricular, Aortic stenosis, Atrial appendage, Isolated atrial amyloidosis, Atrial natriuretic peptide, Atrial natriuretic factor, ATTR, TTR

## Abstract

**Background:**

Recent studies have reported atrial involvement and coexistence of aortic stenosis in transthyretin (ATTR) cardiac amyloidosis (CA). However, pathological reports of extraventricular ATTR amyloid deposits in atrial structures or heart valves are limited, and the clinical implications of ATTR amyloid deposits outside the ventricles are not fully elucidated.

**Case presentation:**

We report 3 cases of extraventricular ATTR amyloid deposits confirmed in surgically resected aortic valves and left atrial structures, all of which were unlikely to have significant ATTR amyloidosis infiltrating the ventricles as determined by multimodality evaluation including ^99m^technetium-pyrophosphate scintigraphy, cardiac magnetic resonance, endomyocardial biopsy and their mid-term clinical course up to 5 years. These findings suggested that these were extraventricular ATTR amyloid deposits localized in the aortic valve and the left atrium.

**Conclusions:**

While long-term observation is required to fully clarify whether these extraventricular ATTR amyloid deposits are truly localized outside the ventricles or are early stages of ATTR-CA infiltrating the ventricles, our 3 cases with multimodality evaluations and mid-term follow up suggest the existence of extraventricular ATTR amyloid deposits localized in the aortic valve and left atrial structures.

## Background

Recent studies have reported atrial involvement and coexistence of aortic stenosis (AS) in transthyretin (ATTR) cardiac amyloidosis (CA) [1–7]. However, pathological reports of extraventricular ATTR amyloid deposits in the valves or in the atrial structures are limited, and the clinical implications of ATTR amyloid deposits outside the ventricles are not fully elucidated.

Herein, we report 3 cases of pathologically diagnosed extraventricular ATTR amyloid deposits in surgically resected aortic valves and left atrial structures, which suggested the existence of extraventricular ATTR amyloid deposits localized in the aortic valve and left atrial (LA) structures.

## Case presentations

### Case 1

**Fig. 1:**
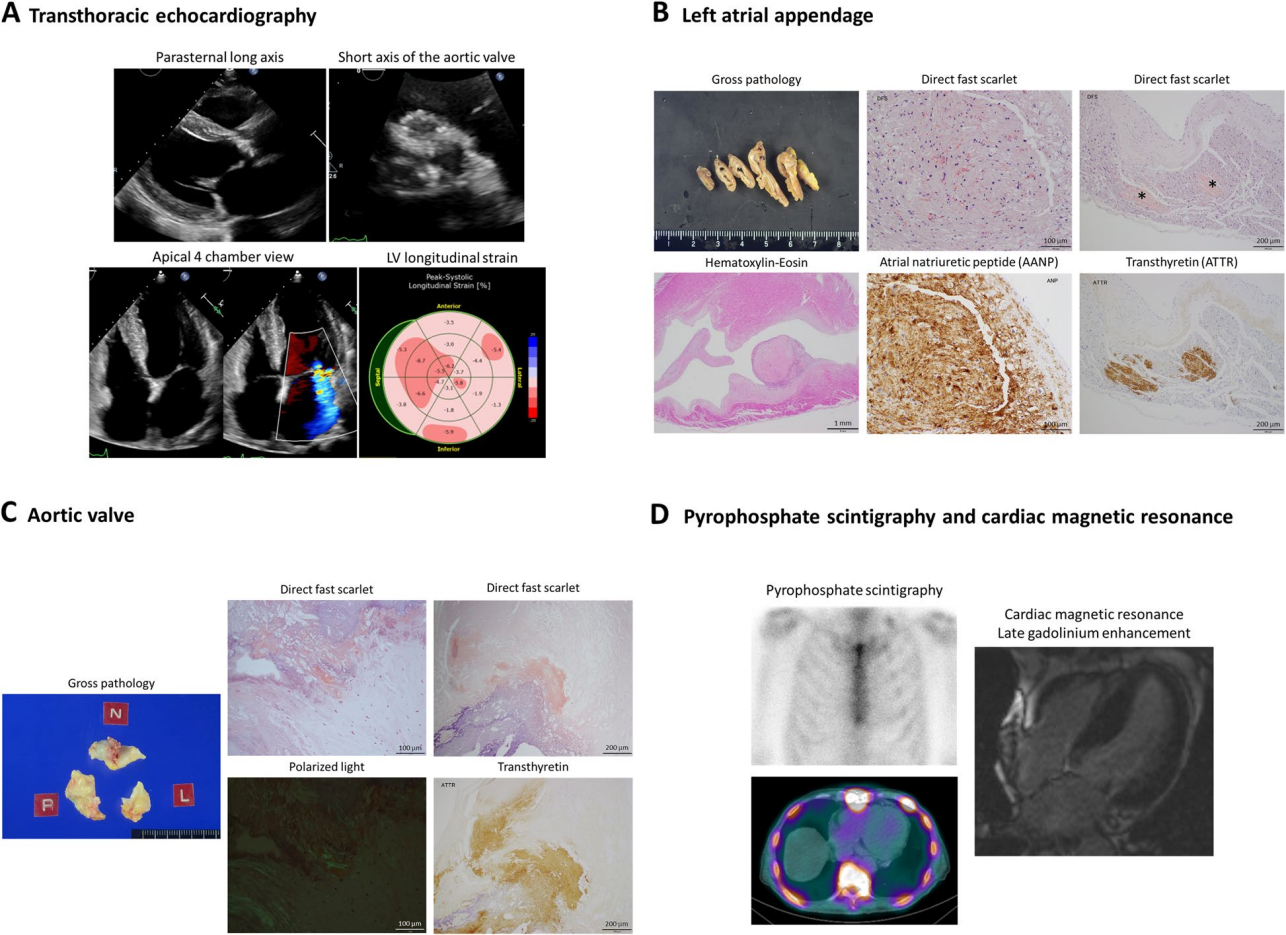
Images of Case 1. **A** Transthoracic echocardiogram showed left ventricular dysfunction (ejection fraction 26%), severe aortic stenosis, and moderate functional mitral regurgitation. **B** Pathology of the left atrial appendage showed diffuse interstitial amyloid deposits by Hematoxylin-Eosin and direct fast scarlet staining, positive for atrial natriuretic peptide immunohistochemical staining, as well as nodular amyloid deposits positive for transthyretin (asterisks). **C** Pathology of the aortic valve revealed nodular amyloid deposits by direct fast scarlet staining with apple-green birefringence under polarized light microscopy, positive for transthyretin immunohistochemical staining. **D** ^99m^Technetium-pyrophophate scintigraphy showed no significant myocardial uptake, and cardiac magnetic resonance showed no late gadolinium enhancement and normal extracellular volume fraction (29.9%)

A 79-year-old man was admitted for acute decompensated heart failure. He had atrial fibrillation (AF, 117 beats/min) and elevated B-type natriuretic peptide (BNP, 1381.7 pg/ml; reference range < 18.4 pg/ml). Echocardiogram showed left ventricular (LV) ejection fraction (EF) 26%, LV end-diastolic/end-systolic dimension (LVEDD/ESD) 57/48 mm, interventricular septum/posterior wall thickness (IVS/PWT) 12/10 mm, global longitudinal strain (GLS) -4.4%, severe AS (peak velocity 4.8 m/s, mean pressure gradient 47 mmHg, aortic valve area (AVA) 0.37 cm^2^) and moderate mitral regurgitation (Fig. 1A). Surgical aortic valve replacement (SAVR), mitral valve repair, Cryo-Maze procedure and LA appendectomy were performed.

Pathology of the resected LA appendage (Fig. 1B) showed diffuse interstitial amyloid deposits positive for atrial natriuretic peptide (AANP) immunohistochemistry, as well as nodular amyloid deposits positive for ATTR immunohistochemistry. Further, pathology of the aortic valve also revealed nodular ATTR amyloid deposits (Fig. 1C). The patient’s postsurgical course was uneventful. ^99m^Technetium-pyrophosphate (^99m^Tc-PYP) scintigraphy showed no atrial or ventricular uptake, and cardiac magnetic resonance (CMR) showed no late gadolinium enhancement (LGE) and normal extracellular volume fraction (29.9%), suggesting that ATTR-CA infiltrating the ventricles was unlikely (Fig. 1D). Troponin T (0.009 ng/ml; reference range < 0.014 ng/ml) and plasma ANP (20.1 pg/ml; reference range < 43.0 pg/ml) were normal. The patient’s EF and GLS improved to 65% and − 17.2%, and BNP decreased to 48.8 pg/ml at 6 months follow up. He has been in NYHA class I and uneventful for 24 months since surgery. No follow up evaluations have suggested ATTR-CA, including latest echocardiogram showing no LV dysfunction or LV hypertrophy (EF 60%, LVEDD/ESD 46/26 mm, IVS/PW 11/11 mm) and low BNP (39.0 pg/ml).

### Case 2

**Fig. 2:**
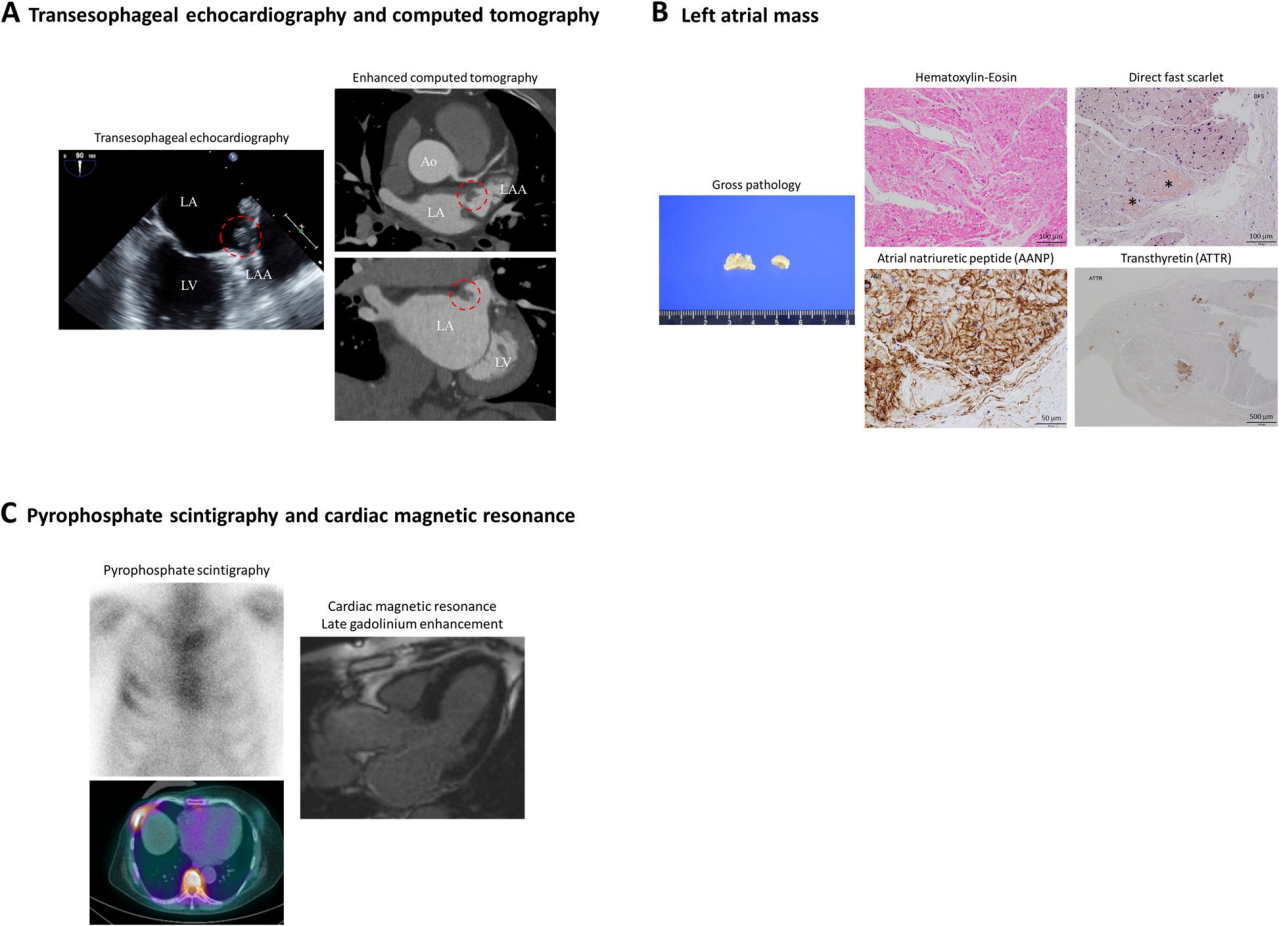
Images of Case 2. **A** Transesophageal echocardiogram and contrast-enhanced computed tomography revealed a 9 mm pedunculated mobile mass on the left atrial wall next to the left atrial appendage. **B** Pathology of the left atrial mass showed diffuse interstitial amyloid deposits (by Hematoxylin-Eosin and direct fast scarlet staining) positive for atrial natriuretic peptide, as well as nodular amyloid deposits positive for transthyretin. **C** ^99m^Technetium-pyrophosphate scintigraphy showed no significant myocardial uptake, and cardiac magnetic resonance showed no ventricular late gadolinium enhancement but slight gadolinium enhancement in the left atrial walls

A 73-year-old woman was admitted for transient ischemic attack. She had no AF, normal echocardiogram (EF 60%, LVEDD/ESD 42/28 mm, IVS/PWT 7/7 mm) and normal BNP (16.5 pg/ml). Transesophageal echocardiogram and contrast-enhanced computed tomography revealed a 9-mm pedunculated mobile mass in the LA next to the LA appendage, which was suspected to be the source of emboli (Fig. 2A). LA mass and partial LA wall resection were performed.

Pathology of the mass showed diffuse interstitial AANP amyloid deposits, as well as nodular ATTR amyloid deposits (Fig. 2B). Troponin T was normal (0.009 ng/ml) and plasma ANP was slightly elevated (66.3 pg/ml). ^99m^Tc-PYP scintigraphy showed no atrial or ventricular uptake, and CMR showed no ventricular LGE but slight enhancement in the LA walls (Fig. 2C). The patient has been uneventful for 24 months since surgery and no follow up data have suggested ATTR-CA; echocardiogram with no LV hypertrophy and normal GLS (EF 64%, LVEDD/ESD 43/28 mm, IVS/PW 8/8 mm, GLS − 20.4%), and normal Trop T (0.012 ng/ml).

### Case 3

**Fig. 3:**
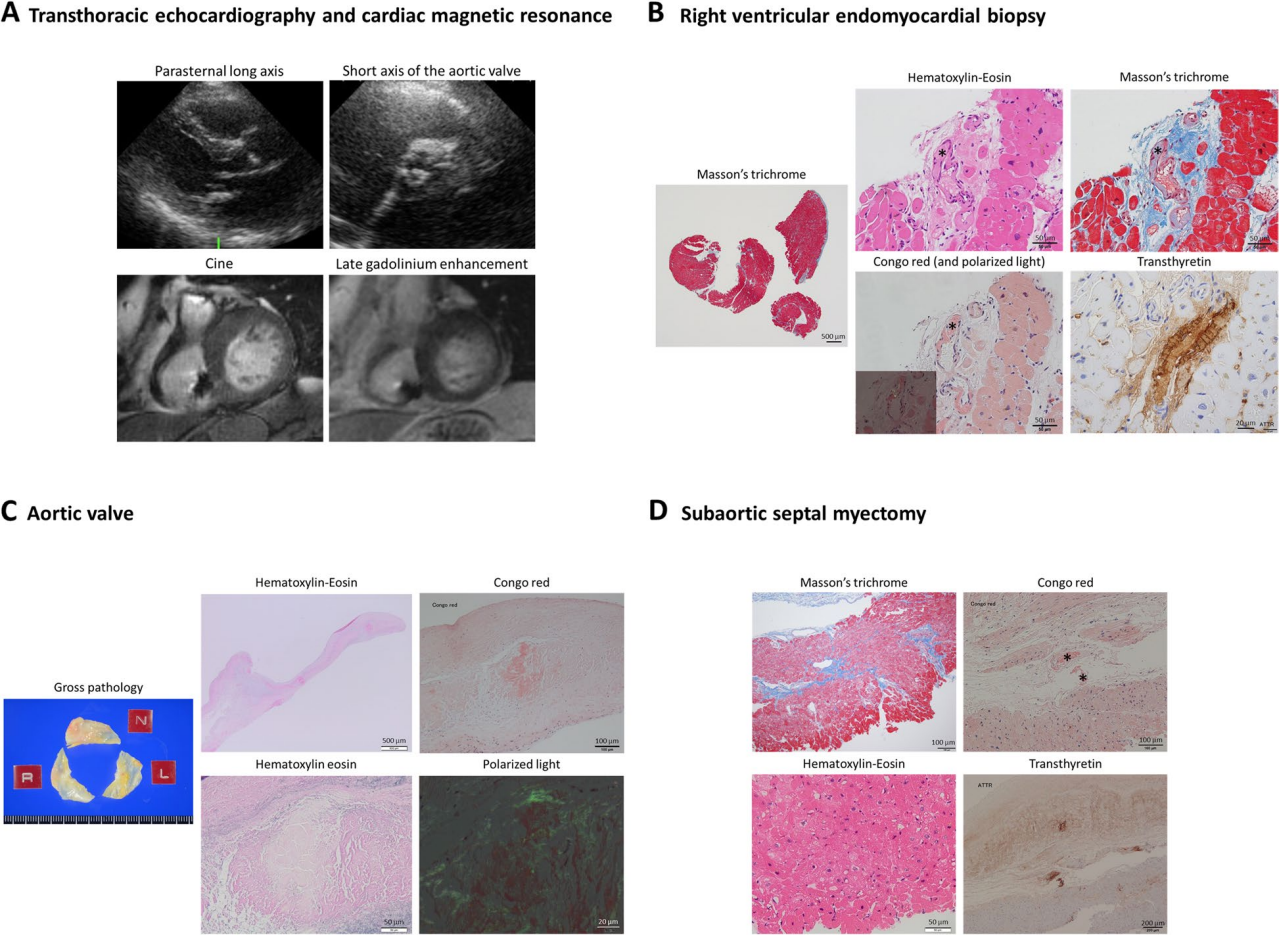
Images of Case 3. **A** Transthoracic echocardiogram showed mild left ventricular hypertrophy and sigmoid septum showing significant left ventricular outflow tract obstruction, with concomitant low gradient aortic stenosis. Cardiac magnetic resonance also showed asymmetric septal hypertrophy (septum thickness 15 mm) with no significant late gadolinium enhancement. **B** Preoperative right ventricular endomyocardial biopsy showed no significant amyloid deposits in the myocardium, except for transthyretin amyloid deposits on the vessel walls of small arteries confirmed by Congo red staining with apple-green birefringence under polarized light microscopy and immunohistochemical staining for transthyretin (asterisks). **C** Pathology of the resected aortic valve showed patchy amyloid deposits confirmed by Congo red staining with apple-green birefringence under polarized light microscopy and immunohistochemical staining positive for transthyretin. **D** Pathology of resected myocardium from subaortic septal myectomy showed no significant amyloid deposits in the myocardium, except for transthyretin amyloid deposits on the vessel walls of small arteries confirmed by Congo red staining and immunohistochemical staining for transthyretin

A 78-year-old woman with a history of AF, pacemaker implantation, long standing hypertension and LV outflow tract obstruction (LVOTO) was admitted for dyspnea on exertion. Troponin T was normal (0.008 ng/ml), and BNP was elevated (261.4 pg/ml). Echocardiogram (Fig. 3A) showed LVEDD/ESD 42/27 mm, IVS/PWT 13/10 mm, LVOTO (provoked peak pressure gradient 92 mmHg) and low gradient moderate AS (peak velocity 2.6 m/s, mean pressure gradient 14 mmHg, AVA 1.23 cm^2^). Perioperative evaluations for LV hypertrophy included CMR showing asymmetric septal hypertrophy (septum thickness 15 mm) with no LGE (Fig. 3A), and right ventricular endomyocardial biopsy (RV-EMB) showing no significant amyloid in the myocardium, except for ATTR amyloid deposits on the vessel walls of small arteries (Fig. 3B). For drug refractory LVOTO with concomitant AS, SAVR and subaortic septal myectomy were performed.

Pathology of the aortic valve showed patchy ATTR amyloid deposits (Fig. 3C). The myocardium from subaortic myectomy showed no significant myocardial amyloid deposits, except for ATTR amyloid on the vessel walls of the small arteries (Fig. 3D), similar to the preoperative RV-EMB. The patient has been followed up for 5 years since surgery and no follow up evaluations have suggested ATTR-CA infiltrating the ventricles; mildly elevated BNP (407.7 pg/ml) but no LV dysfunction or LV hypertrophy on echocardiogram (EF 60%, LVEDD/ESD 40/25 mm, IVS/PW 10/10 mm at 5 years).

## Discussion and conclusions

We report 3 cases of extraventricular ATTR amyloid deposits confirmed in surgically resected aortic valves and LA structures, all of which were unlikely to have ATTR amyloidosis infiltrating the ventricles as determined by multimodality evaluation and mid-term clinical course up to 5 years (summarized in Table 1). Our cases suggest the existence of extraventricular ATTR amyloid deposits localized in the aortic valve and LA structures.

**Table 1:** Summary of cases with extraventricular transthyretin amyloid deposits

	Case 1	Case 2	Case 3
**Patients’ characteristics**			
Age and sex	79 yo male	73 yo female	78 yo female
Clinical diagnosis	Severe AS Atrial fibrillation LV dysfunction Functional MR	Transient ischemic attack LA mobile mass	AS LVOT obstruction
History of carpal tunnel syndrome or lumber canal stenosis	No	No	No
Surgical interventions	Aortic valve replacement Mitral valve plasty Cryo-Maze LA appendage resection	LA mass and partial LA wall resection	Aortic valve replacement Subaortic septal myectomy
**Amyloid deposition and types**			
Left atrium / left atrial appendage	AANP + ATTR	AANP + ATTR	n/a
Aortic valve	ATTR	n/a	ATTR
Ventricles	Unlikely by PYP scintigraphy and CMR	Unlikely by PYP scintigraphy and CMR	Non-significant (ATTR limited to vessel walls of both ventricles)
**Post surgical course**			
	• Normalized ejection fraction and significantly decreased BNP at 6 months • Uneventful for 24 months with no echocardiogram findings suggesting ventricular cardiac amyloidosis	• Normal ventricular function • Uneventful for 24 months, with normal troponin and no echocardiogram findings suggesting ventricular cardiac amyloidosis	• No heart failure events and no echocardiogram findings suggesting ventricular cardiac amyloidosis for 5 years

*AANP* Atrial natriuretic peptide, *AS* Aortic stenosis, *BNP* B-type natriuretic peptide, *CMR* Cardiac magnetic resonance, *LA* Left atrium, *LV* Left ventricle, *LVOT* Left ventricular outflow tract, *MR* Mitral regurgitation, *PYP* Pyrophosphate, *ATTR* Transthyretin

### AANP derived and ATTR derived amyloid deposits in the atrium

AANP amyloid deposits in the atrium, often referred to as isolated atrial amyloidosis (IAA) [8, 9], have been reported in surgically resected appendages and autopsy studies [4, 10–16]; however, very few histologically reports of ATTR amyloid in the atrium are published [4, 10–12]. Further, most previous studies were limited to evaluation of the atrium and did not evaluate the ventricles or other cardiac structures. Thus, the relationship between ATTR amyloid deposits in the atrium and ATTR-CA infiltrating the ventricles, and the clinical impact of atrial ATTR amyloid deposits remains unclear.

Di Bella et al. reported in 2018 that atrial involvement in ATTR-CA is an early stage of ATTR-CA [7]. However, recent reports show contradictory results [2, 4]. Ichimata et al. [4], in line with Di Bella et al., reported in a study of 44 autopsy cases of ATTR-CA evaluating multiple cardiac regions that atria-predominant ATTR amyloid deposition may precede TTR amyloidosis of the ventricles; however, by using cluster analysis from retrospective insights. On the other hand, Hussain et al. [2] recently reported in a study of 580 patients with ^99m^Tc-PYP scintigraphy that 58 patients had atrial uptake without ventricular uptake, while 105 patients had ventricular uptake without atrial uptake, indicating that atrial involvement may not necessarily precede ventricular involvement.

As ATTR amyloids are believed to be derived and formed from circulating transthyretin protein synthesized by the liver, ATTR amyloid deposits in the atrium may be a part of systemic disease and ATTR amyloid deposits could be found in other tissue sites after long-term follow up. However, our findings support the possibility that they could be localized atrial ATTR amyloid deposits. While the clinical significance of atrial ATTR amyloid deposits has not been fully elucidated and long-term close follow-up may be justified at this point, our 2 cases of surgically resected ATTR amyloid deposits in the atrium with mid-term prospective follow up, the first to be reported with multimodality evaluations, suggest the possibility of extraventricular ATTR amyloid deposits localized in the atrium.

### ATTR amyloid deposits in the aortic valve

While various types of amyloid deposits including light-chain (AL), amyloid A protein (AA) and Apo-AI have been reported in surgically resected aortic valves and autopsy studies, very few histological reports of ATTR amyloid in the aortic valve have been published [17–19], and their relationship with ATTR-CA infiltrating the ventricles is not fully understood. Recent studies have reported that ATTR-CA (infiltrating the ventricles) coexists in 11–16% of elderly patients with AS undergoing transcatheter aortic valve implantation [5, 6]. However, histological diagnosis is rarely confirmed in these patients, and the association among ATTR-CA, AS and aging has not been clarified.

Recently, Singal et al. studied 46 surgically resected aortic valves with concomitant ventricular biopsy and reported that 33 (71.7%) had aortic valvular amyloid deposits (including 19 ATTR derived); however, none had amyloid deposits in the ventricle, and they concluded that the majority were “isolated valvular ATTR amyloid deposits” [19]. In line with their report, our 2 surgical cases of ATTR amyloid deposits in the aortic valve with longer follow-up (up to 5 years) and multimodality evaluations support the possibility of localized ATTR amyloid deposits in the aortic valves.

Similar to the discussion with ATTR amyloid deposits in the atrium, deposition of ATTR amyloid is believed to be a systemic process, thus we could not rule out the possibility that valvular ATTR amyloid deposit is an early stage of ATTR-CA (infiltrating the ventricles) without long-term follow up. However, our cases suggest the possibility that they could be localized valvular ATTR amyloid deposits. Our findings also suggests that, to understand the pathophysiology of patients with concomitant AS and ATTR-CA, the existence of amyloid deposits localized in the valves should be taken into consideration.

### Patient perspectives

While clinical implications of extraventricular ATTR amyloid deposits localized outside the ventricles are not fully elucidated, our cases are the first detailed report of surgically resected cases and with mid-term follow up (up to 5 years), which suggested the existence of extraventricular ATTR amyloid deposits localized in the aortic valve and the atrial structures. Diagnosis of amyloid deposits were confirmed by Congo red or direct fast scarlet staining, followed by immunohistochemistry staining for ATTR and AANP. Mass spectrometry or immunoelectron microscopy was unavailable in our patients, which might have provided further insights because of their proven superior sensitivity compared to immunohistochemistry [20, 21].

It could be hypothesized that our cases were localized amyloid deposits rather than an early ATTR-CA, and raises the possibility of localized “innocent” deposits not strictly related to systemic deposition as reported in literatures [10, 22–24]. However, non-cardiac manifestations or extracardiac ATTR amyloid deposits as in the case of carpal tunnel syndrome [25, 26] are believed to precede ATTR-CA by a period of years (although none of our 3 cases had history of carpal tunnel syndrome or lumber canal stenosis). Further long-term prospective studies will clarify the clinical implications of extraventricular ATTR amyloid deposits and help us understand whether they are useful for early diagnosis and treatment intervention of ATTR-CA [27].

## Data Availability

Data associated with this manuscript are not publicly available, but can be made available by the corresponding author upon reasonable request.

## Abbreviations

ANP: Atrial natriuretic peptide
AS: Aortic stenosis
CA: Cardiac amyloidosis
CMR: Cardiac magnetic resonance
EMB: Endomyocardial biopsy
IAA: Isolated atrial amyloidosis
LA: Left atrium
SAVR: Surgical aortic valve replacement
^99m^Tc-PYP: ^99m^Technetium-pyrophosphate
ATTR: Transthyretin
